# Anti-inflammatory activity of the ethanol extract of *Dictamnus dasycarpus* leaf in lipopolysaccharide-activated macrophages

**DOI:** 10.1186/1472-6882-14-330

**Published:** 2014-09-03

**Authors:** Chiranjit Ghosh, Bora Hong, Subhasis Batabyal, Tae-Il Jeon, Seung-Hak Yang, Seong Gu Hwang

**Affiliations:** Department of Microbiology and Cell Biology, Indian Institute of Science, Bangalore, 560 012 India; Department of Animal Life and Environmental Science, Hankyong National University, Anseong, 456-749 Republic of Korea; Department of Veterinary Biochemistry, West Bengal University of Animal and Fishery Sciences, Kolkata, India; Department of Animal Science, College of Agriculture & Life Sciences, Chonnam National University, Gwangju, Republic of Korea; Animal Environment Division, National Institute of Animal Science, R.D.A., Suwon, 441-706 Republic of Korea

**Keywords:** *Dictamnus dasycarpus*, NF-κB, Nitric oxide, Cytokines, Macrophages, Inflammation

## Abstract

**Background:**

*Dictamnus dasycarpus* is widely used as a traditional remedy for the treatment of eczema, rheumatism, and other inflammatory diseases in Asia. The current study investigates the molecular mechanism of anti-inflammatory action of the ethanol extract of *Dictamnus dasycarpus* leaf (DE) in lipopolysaccharide (LPS)-stimulated RAW 264.7 cells.

**Methods:**

Nitric oxide (NO) production was assessed by Griess reaction and the mRNA and protein expressions of pro inflammatory cytokines, transcription factor, and enzymes were determined by real-time RT-PCR and immunoblotting analysis.

**Results:**

DE (0.5 and 1 mg/mL) suppressed the NO production by 10 and 33%, respectively, compared to the untreated group in LPS-stimulated RAW 264.7 cells. DE (0.5 and 1 mg/mL) reduced the mRNA expression of key transcription factor nuclear factor-κB by 7 and 24%, respectively compared to the untreated group in LPS activated macrophage. The pro inflammatory cytokines such as tumor necrosis factor α and interleukin 1β were also decreased by DE treatment. Moreover, the protein expression of pro inflammatory enzymes, inducible nitric oxide synthase and cyclooxygenase 2 were also dramatically attenuated by DE in a dose dependent manner.

**Conclusions:**

These results suggest that *Dictamnus dasycarpus* leaf has a potent anti-inflammatory activity and can be used for the development of new anti-inflammatory agents.

## Background

Inflammation is a crucial defense mechanism of organisms against invading pathogens and tissue injury; however, chronic inflammation can lead to several diseases such as atherosclerosis, obesity, neurodegenerative diseases, bronchitis, and allergies [1–4]. The discovery of herbal anti-inflammatory compounds and understanding their mode of action would contribute to the development of new therapies against chronic inflammation and its associated disorders.

Inflammation is a complex process involving a network of cytokines responsible for the expression of many proinflammatory genes. Nuclear factor-kappa B (NF-κB) is considered as a key transcriptional regulator of several genes involved in immune and inflammatory responses [5]. NF-κB is a heterotrimeric complex composed of p50, p65, and IκB-α, and its activation is the key event of many proinflammatory transcriptional programs that finally lead to the development of a variety of pathological disorders. NF-κB has a role in the transcription of several proinflammatory mediators such as inducible nitric oxide synthase (iNOS), cyclooxygenase 2 (COX-2), tumor necrosis factor alpha (TNF-α), and interleukin (IL)-1β [6–8]. Nitric oxide (NO) is synthesized by the oxidative deamination of ʟ-arginine by iNOS. Although, NO plays an important role in host defense mechanism but excessive production of NO and its reactive nitrogen intermediates is involved in the pathogenesis of several chronic diseases [9]. Biologically, the post-receptor events of TNF-α and IL-1β are closely related, but their structures and receptors are different; TNF-α and IL-1β act synergistically to induce the expression of several major proinflammatory mediators such as prostaglandins, leukotrienes, and NO [10]. IL-1β stimulates the transcription of COX-2, an enzyme that plays an important role in the regulation of inflammation by inducing the production of prostaglandins [11].

*Dictamnus dasycarpus* Turcz is a perennial herbal plant widely distributed in Asia. The root bark of this plant has been widely used as a traditional remedy for the management of various ailments such as jaundice, eczema, rubella, scabies, rheumatism, cold, and headache [12, 13]. Many biologically active components have been identified from the root bark of *D. dasycarpus* such as limonoids, furoquinoline alkaloids, flavonoids, coumarins, sesquiterpenes, sesquiterpene glycosides, and phenolic glycosides [14–17]. To date, however, the anti-inflammatory effects of the leaf extract of this plant have not been studied. Therefore, in the present work, we investigated the anti-inflammatory activity of the ethanolic leaf extract of *D. dasycarpus* in RAW 264.7 cell lines stimulated by lipopolysaccharide (LPS).

## Methods

### Reagents

Cell culture reagents were obtained from GIBCO/Invitrogen (Carlsbad, CA). LPS was acquired from Sigma–Aldrich (St. Louis, MO). Primers were obtained from Macrogen (Korea). Antibodies against iNOS, COX-2, and β-actin were obtained from Santa Cruz (Santa Cruz, CA).

### Preparation of plant extracts

Locally purchased *D. dasycarpus* leaves were freeze dried and converted to powder form. The plant material was authenticated by Professor Tae Wan Kim, Department of Plant and Environmental Science, Hankyong National University, Republic of Korea. The voucher number of the plant is HKNU#201203 deposited at the herbarium of Plant Extract Bank at Hankyong National University for future reference. The powder was mixed with 80% ethanol for 24 h, filtered (No. 4 Whatman paper; GE Healthcare, UK), and vacuum evaporated. The extract was then subjected to freeze drying, after that it was mixed with the cell culture medium.

### Cell line

RAW 264.7, a macrophage-like cell line, was obtained from the American Type Culture Collection (Manassas, VA, USA) and cultured in Dulbecco’s modified Eagle’s medium supplemented with 10% heat-inactivated fetal bovine serum, 100 U/mL penicillin, and 100 μg/mL streptomycin and maintained at 37°C in a 5% CO_2_ and 95% air (CO_2_ incubator; Heal Force).

### Cell viability assay

Cell viability was determined using a Cell Counting Kit (CCK)-8 (Dojindo Laboratories, Japan), according to the manufacturer’s instruction. Briefly, RAW 264.7 cells were treated with different concentrations of DE (0.5 and 1 mg/mL) for 1 h before the addition of the LPS. After 24 h the medium containing the test compounds was replaced by medium containing 10 μL CCK-8 reagents and incubated in the dark for 2 h. The amount of formazan dye was measured by absorbance at 450 nm using an enzyme-linked immunosorbent assay (ELISA) microplate reader (Tecan Infinite F50). Each assay was carried out in triplicate.

### Nitrite determination

RAW 264.7 cells (2.0 × 10^5^ cells/well) were seeded in 96-well plates and allowed to adhere overnight. Then, the medium was removed and replaced with 0.2 mL fresh medium with or without different concentrations of DE (0.5 and 1 mg/mL). After 1-h incubation, LPS stimulation was performed at 1 μg/mL for 24 h. The cell-free culture medium was collected and 50 μL were used for NO determination. The nitrite accumulated in the culture medium was measured as an indicator of NO production based on the Griess reaction [18]. Briefly, 50 μL cell culture medium were mixed with an equal volume of the Griess reagent (equal volumes of 1% (w/v) sulfanilamide in 5% (v/v) phosphoric acid and 0.1% (w/v) naphthylethylenediamide-HCl), incubated at room temperature for 15 min, and the absorbance was measured at 540 nm using an ELISA microplate reader (Tecan Infinite F50). The amount of nitrite present in the samples was calculated by means of a standard curve generated using serial dilutions of NaNO_2_ in fresh culture medium.

### RNA extraction and cDNA synthesis

The cells were seeded in 6-well plates (1.0 × 10^6^ cells/well) and incubated with or without DE (0.5 and 1 mg/mL) prior to LPS stimulation (1 μg/mL). After 24 h, total RNA was isolated from the cells using the RNAiso Plus reagent (Takara, Japan). One microgram of RNA was reverse transcribed using a High Capacity cDNA Reverse Transcription Kit (Applied Biosystems, USA) to obtain cDNA according to the manufacturer’s protocol. Briefly, a total reaction volume of 20 μL was incubated as follows in a TC-E-96G Gene Pro (Bioer Technology, China): 10 min at 25°C, 120 min at 37°C, 5 min at 85°C, and holding at 4°C.

### Real-time PCR

Real-time PCR was performed with an Applied Biosystems 7300 Real-time PCR System (Applied Biosystems, USA) using Power SYBR® Green PCR Master Mix (Applied Biosystems, USA) according to the manufacturer’s protocol. Briefly, PCR was performed in a final volume of 20 μL including 10 ng sample cDNA, 5 μM specific forward and reverse primers, and 10 μL Power SYBR® Green PCR Master Mix. PCR reactions consisted of an initial denaturing cycle at 95°C for 10 min, followed by 40 amplification cycles of 15 s at 95°C and 1 min at 60°C. The primers were purchased from Macrogen (Seoul, Korea). Gene-specific primers are shown in Table 1. The expression levels of TNF-α, IL-1β, and NF-κB were normalized using β-actin as an internal control. Analysis was carried out in triplicate.

**Table 1:** **Primer used in real time PCR**

Target gene	PCR primer sequences (5′-3′)
TNF-*α*	F-GCT GAG CTC AAA CCC TGG TA
	R-CGG ACT CCG CAA AGT CTA AG
IL-lβ	F-TGCf AGA GTT CCT ACA TGG TCA ACC C
	R-GTG CTG CCT AAT GTC CCC TTG AAT C
p65-NF-_K_B	F-ACC TGG AGC AAG CCA TTA GC
	R-CGG ACC GCA TTC AAG TCA TA
β-actin	F-CAC CCC AGC CAT GTA CGT
	R-GTC CAG ACG CAG GAT GGC

### Western blot analysis

The cells were seeded in 6-well plates (1.0 × 10^6^ cells/well) and incubated with DE (0.5 and 1 mg/mL) prior to LPS stimulation (1 μg/mL). After 24 h, cell extracts were prepared by adding a protein extraction solution (iNtRON Biotechnology). The lysates were clarified by centrifugation at 28000 × *g* for 15 min at 4°C and the protein content of the supernatants was determined using a modified Bradford assay. The protein samples (30 μg) were separated by sodium dodecyl sulfate-polyacrylamide gel electrophoresis (SDS-PAGE) and transferred to nitrocellulose membranes (Schleicher &Schuell Bioscience GmbH). The membranes were blocked with 5% skim milk and probed with the following primary antibodies: COX-2 (Santa Cruz); iNOS (Santa Cruz), and β-actin (Santa Cruz). The specific proteins were identified by further incubation of the corresponding membranes with horseradish peroxidase-conjugated secondary antibodies followed by treatment with enhanced chemiluminescence reagent (Chromogen) for 5 min and exposed to radiographic film (Kodak) for 1–10 min.

### Statistical analysis

All quantitative data are representative of at least three independent experiments. Quantitative data are presented as the mean ± standard deviation (SD), and analysis of the variance was used to calculate the statistical significance of the differences using Duncan’s test for multiple comparisons of the observed means (P < 0.05) using SAS 9.2 statistical software.

## Results and discussion

### Effects of DE on the viability of LPS-stimulated RAW 264.7 cells

In this study, we investigated the cytotoxic effect of DE on LPS-stimulated RAW 264.7 cells using the CCK-8 assay. The cells were treated with LPS (1 μg/mL) and incubated for 24 h in the presence or absence of DE (0.5 and 1 mg/mL). The concentration of DE and the duration of DE treatment used in this study had no significant effect on the viability of RAW 264.7 cells (Data not shown). Therefore, these concentrations of DE were chosen for further experiments.

### Effects of DE on NO production in LPS-stimulated RAW 264.7 cells

**Figure 1:**
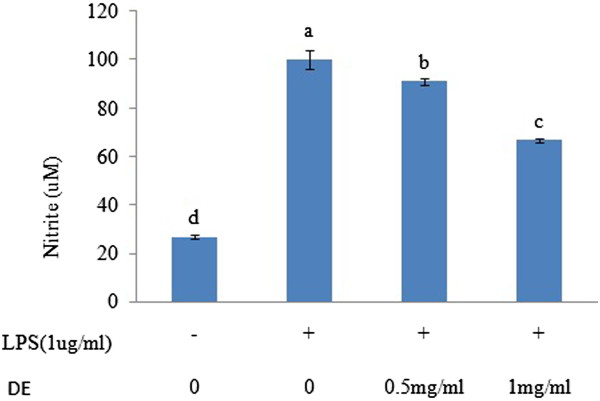
**Effects of DE on NO production.** RAW 264.7 cells were pretreated with different concentrations of DE (0, 0.5 and 1 mg/mL) and then treated with LPS (1 μg/mL). The medium was harvested 24 h later and assayed for nitrate production. Data are the mean ± SD of at least three experiments. Bars within the same panel with different letters are significantly different (*P < 0.05*).

NO is a highly unstable gas required for immune regulation, platelet inhibition, and neurotransmission; however, excessive production of NO, especially in macrophages, is associated with the pathogenesis of several diseases such as cytotoxicity, cancer, and autoimmune disorders [7]. NO is synthesized due to the oxidative deamination of ʟ-arginine by iNOS [19]. Macrophages are distributed throughout the body and play a crucial role in both host defense mechanisms and inflammation. LPS is an endotoxin isolated from bacteria, and stimulation of macrophages with LPS results in the high production of NO by iNOS [20]. As shown in Figure 1, DE suppressed the production of NO in a dose-dependent manner by 10 and 33%, respectively, compared to the untreated group indicating its anti-inflammatory characteristics.

### Effects of DE on the mRNA expression of TNF-α, IL-1β, and NF-κB in LPS-stimulated RAW 264.7 cells

**Figure 2:**
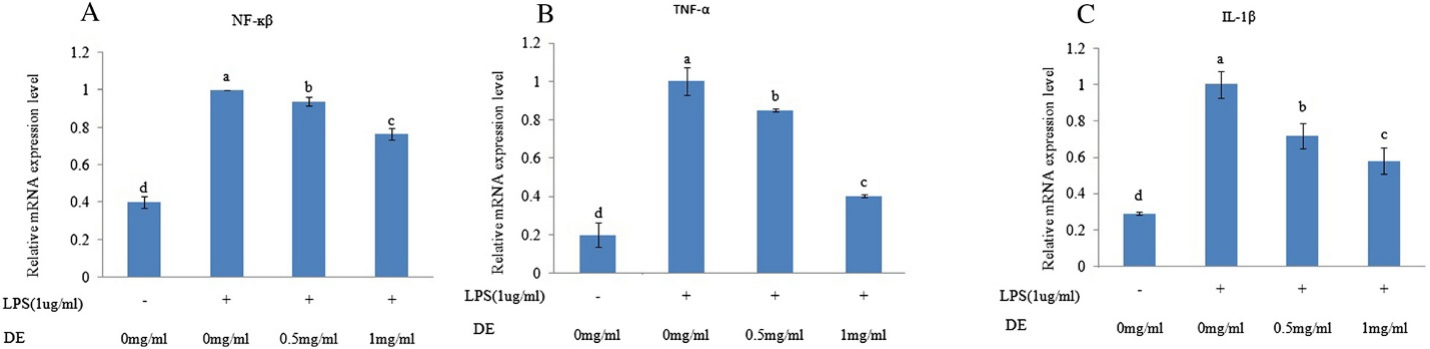
**Effects of DE on the mRNA expression of TNF-α, IL-1β, and NF-κB in LPS-stimulated RAW 264.7 cells.** RAW 264.7 cells were pretreated with or without DE (0, 0.5 and 1 mg/mL) and then treated with LPS (1 μg/mL). After 24 h incubation, total cellular RNA was isolated from the cells. The expression levels of **(A)** NF-κB, **(B)** TNF-α, and **(C)** IL-1β were quantified by real-time PCR. The expression of each gene was normalized using β-actin as an internal control. Each bar represents the mean ± SD of three independent experiments. Bars within the same panel with different letters are significantly different (*P < 0.05*).

Macrophages are major immune cells that play an important role in the inflammatory response. LPS stimulates toll-like receptor 4 on the surface of macrophages, which induces the activation of the NF-κB signaling pathway [21]. NF-κB is an important transcription factor and its activation plays a vital role in the secretion of various proinflammatory mediators such as iNOS, COX-2, TNF-α, and IL-1β. NF-κB is a heterotrimeric complex composed of p50, p65, and IκB-α; the degradation of IκB and its nuclear translocation are considered to be crucial for NF-κB activation [5]. It has been reported that p65 serves as a major component in the activation of NF-κB in LPS-stimulated RAW 264.7 cells [22]. To investigate the effect of DE on LPS-induced NF-κB activation, we examined the effect of DE on p65 nuclear translocation. As shown in Figure 2A, DE significantly and dose-dependently inhibited LPS-induced NF-κB activation by 7 and 24%, respectively, which suggests that the anti-inflammatory effect of DE is partly mediated by blocking NF-κB activation. TNF-α and IL-1β act synergistically to induce the expression of several major proinflammatory mediators such as prostaglandins, leukotrienes, and NO. Previous studies showed that specific blockade of TNF-α and IL-1β reduces the severity of inflammation. IL-1β and TNF-α serve as potent inducers of the expression of various inflammation-related genes, resulting in the amplification of inflammation [12]. As shown in Figure 2B and C, LPS substantially increased the production of TNF-α and IL-1β. However, DE significantly inhibited the LPS-induced production of TNF-α and IL-1β in a concentration-dependent manner in RAW 264.7 cells.

### Effects of DE on the expression of COX-2 and iNOS in LPS-stimulated RAW 264.7 cells

**Figure 3:**
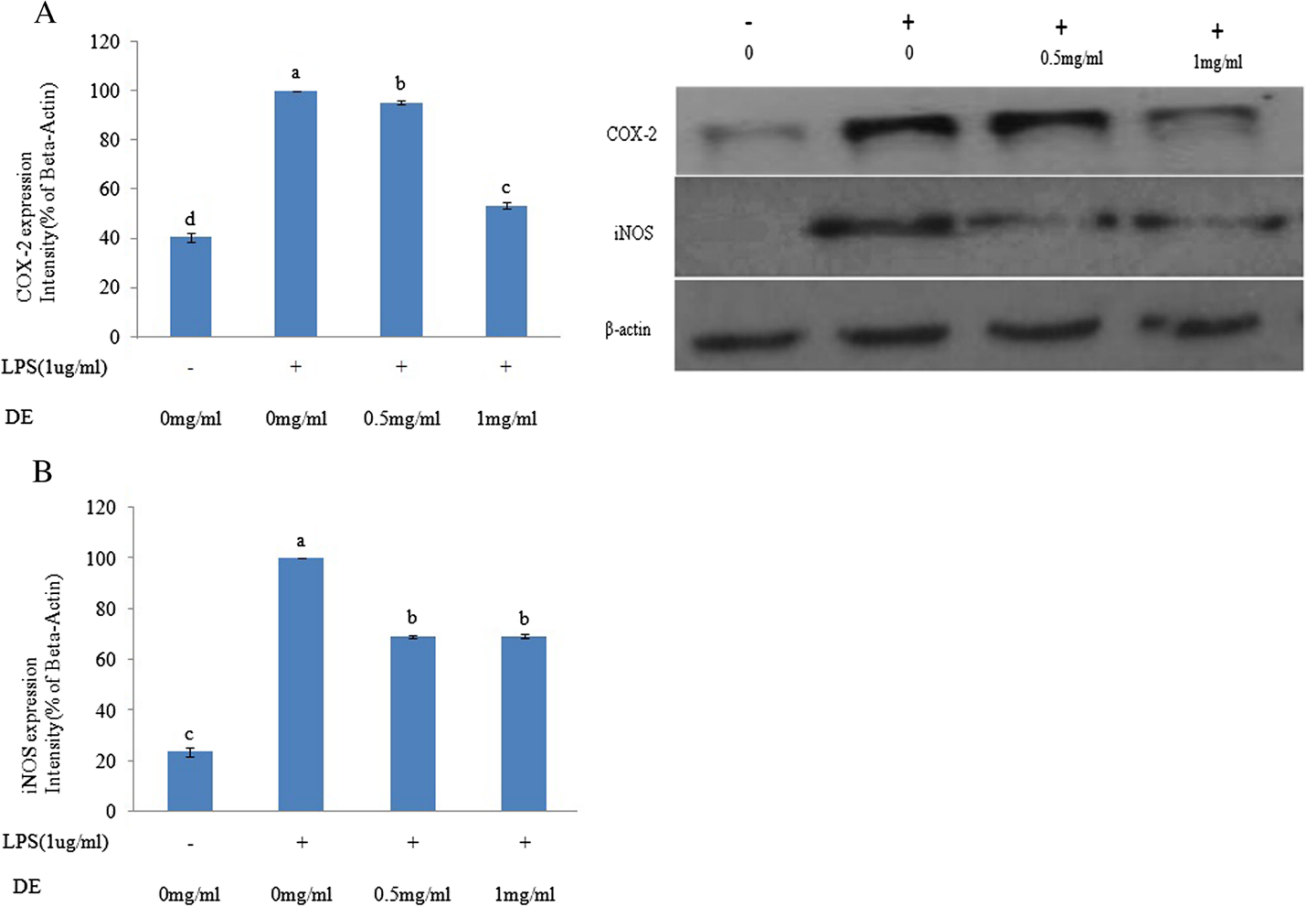
**Effects of DE on the protein expression of COX-2 and iNOS in LPS-stimulated RAW 264.7 cells.** RAW 264.7 cells were pretreated with or without DE (0, 0.5 and 1 mg/mL) and then treated with LPS (1 μg/mL). After 24 h incubation, cell lysates (30 μg/mL) were separated by SDS-PAGE, transferred to a nitrocellulose membrane, and blotted with anti-COX-2, anti-iNOS, and β-actin antibodies. Quantification of COX-2 and iNOS protein expression was performed by densitometric analysis. β-Actin was used as an internal control. The values are expressed as a percentage of the maximal band intensity in the culture treated with LPS alone. Data are the mean ± SD of COX-2 / β-actin **(A)** and iNOS / β-actin **(B)** of at least three separate experiments. Bars within the same panel with different letters are significantly different (*P <0.05*).

COX-2 and iNOS plays an important role in the expression of proinflammatory mediators such as NO and prostaglandin E2 at inflammatory sites. The iNOS and COX-2 gene promoters contain homologous consensus sequences for the binding of NF-κB, which stimulates the activation of iNOS and COX-2 gene transcription [23]. NO is produced from the oxidation of the amino acid arginine by the NOS family. Among the three isoforms of NOS, endothelial NOS and neuronal NOS are constitutively expressed, whereas iNOS is mainly generated by stimulated macrophages and its overproduction is associated with the pathogenesis of several inflammation-related diseases, including cancer [7]. It has been noted that the induction of COX-2 is closely related to NO production and its overproduction has been linked to the development of inflammation and carcinogenesis [24]. As shown in Figure 3, LPS substantially increased the production of COX-2 and iNOS and DE significantly inhibited the LPS-induced production of COX-2 and iNOS in a concentration-dependent manner in RAW 264.7 cells indicating its anti-inflammatory characteristics.

## Conclusions

In the present study, we demonstrated that DE significantly and dose-dependently inhibited the expression of COX-2 and iNOS, which inhibited NO production by reducing NF-κB expression. Moreover, the inhibition of TNF-α and IL-1β by DE is mediated by blocking NF-κB activation in RAW 264.7 cells. Therefore, these results suggest that DE can be useful for the amelioration of various inflammation-related diseases and can be a potential herbal component for the development of new anti-inflammatory drugs.
